# Supplementary material for: Are carbon emissions trading and green financial instruments synergistic? -Comprehensive quantitative research based on content analysis
Source: PLoS One. 2024 Mar 7;19(3):e0298601. doi: 10.1371/journal.pone.0298601 (PMC10919601; doi:10.1371/journal.pone.0298601)
Supplement: S1 Data — (ZIP) [file pone.0298601.s001.zip › Data/地级市Z_F工作报告.zip!/║■─╧/║Γ╤⌠╩╨/└┤╫╘╣1⁄2╓┌║┼í╛┬φ┐╦╩2╛▌═°í┐_2016.docx]

各位代表：

现在，我代表市人民政府，向大会报告政府工作，请予审议，并请各位政协委员和其他列席人员提出意见。

一、“十二五”时期及2015年工作回顾

五年来，在党中央、国务院和省委、省政府及市委的正确领导下，全市上下主动适应新常态、积极应对大环境、着力营造小气候，突出稳增长、促改革、调结构、惠民生、防风险，总体完成了“十二五”规划的主要目标任务，为全面建成小康社会奠定了坚实基础。

这五年，经济平稳快速发展，综合实力跃上新台阶。2015年，全市地区生产总值、财政总收入分别达到2600、261.86亿元(预计数，下同)，分别是2010年的1.83、2.31倍，同比分别增长8.8%、11.1%，人均GDP突破5000美元，综合实力稳居全省第一方阵；银行业金融机构存、贷款余额分别为2787.27、1445.77亿元，存贷比优化为51.9%，比2010年提高11.2个百分点；城乡居民人均可支配收入分别为26514、14500元，分别是2010年的1.71、2.01倍，人民生活正向全面小康大步迈进。

这五年，项目建设扎实推进，发展基础大力夯实。项目数量和投资规模连创新高，固定资产投资年均增长30.9%。实施重点项目1728个，完成投资3506亿元，分别是“十一五”时期的2.7、3.93倍，富士康、中国五矿等20家世界500强和18家央企来衡发展。融资平台不断夯实，设立和引进金融机构68家；市级投融资公司现代企业制度基本建立，2015年到位融资251.9亿元，是2010年的23.7倍。园区平台实现历史性突破，创新创业园区“135”工程和“五大平台”建设扎实推进，获批国家级湘南承接产业转移示范区、国家级高新区和全省第一家综保区。2015年，省级及以上产业园区规模工业增加值占全市规模工业增加值的比重达61.6%，比2010年提高36个百分点。

这五年，区域统筹不断加强，城乡面貌发生深刻变化。城市骨架全面拉开，“1189”立体交通基本成形，铁路、高速公路总里程分别跃居全省第2、第3位，南岳机场平均客座率在全国新开支线机场中处于领先地位；大力推进来雁新城、滨江新区等一批城市地标项目建设，中心城区辐射和带动能力大幅提升；城区、县城、集镇和新农村建设全面协调发展，全市城镇化率达49.2%。以创建全国文明城市为统揽的“八城同创”扎实推进，城市管理体制改革深入开展，城市管理水平实现质的提升；统筹推进城乡环境同治，中心集镇“五乱”现象得到有效遏制，全市人民的归属感和幸福感明显增强。

这五年，绿色衡阳加快建设，生态环境明显改善。获批国家生态文明先行示范区。圆满完成湘江流域保护和治理第一个“三年行动计划”，水污染、重金属污染、大气污染整治取得明显成效，县级以上城镇生活污水处理率达85.6%。节能减排目标全面完成，六大高耗能行业比重比2010年下降6.9个百分点。扎实推进通道绿化和乡村、裸露山地造林绿化，全市森林覆盖率达46.3%，衡阳的天更蓝、水更清、地更绿。

这五年，社会事业全面进步，人民群众得到更多实惠。坚持财政收入主要用于民生的导向，民生投入累计达1200.76亿元，年均增长24.5%，全面或超额完成127件重点民生实事。强化问题导向，抓实底线工作，安全生产考核连续3年名列全省第一，信访工作连续5年获评全省先进，社会大局稳定和谐。公共服务体系加快完善，社会保障体系不断健全并向农村延伸。农网升级改造、中心城区商品房产权办理遗留问题、安置房建设等“三最”问题逐步得以解决。

各位代表，刚刚过去的2015年，在全市上下共同努力下，我们总体完成了市十四届人大四次会议确定的主要目标任务，开创了经济社会发展的新局面。一年来，主要抓了以下工作：

一是突出抓产业发展。三次产业结构优化为15.2∶44.7∶40.1，第三产业占比提升2.1个百分点。全年实现高新技术产业增加值358亿元，增长18%。新型工业加速推进。克服清理历史虚增影响，规模工业增加值增速从4月份触底企稳后连续8个月回升。市直“两区一园”完成调规扩区，衡山科学城启动一期建设。水口山经开区获批国家循环化改造示范试点园区，中国五矿(湖南)铜业获批国家工业生态设计示范企业。现代农业加速转型。粮食总产量实现“十二连增”。“一流转五服务”工作深入推进，流转土地507.78万亩。我市成为全国油茶产业发展示范市、全国农业农村信息化示范基地，衡南获批国家现代农业示范区，祁东黄花菜获评国家地理标志产品。现代服务业加速壮大。基本完成国家服务业综合改革试点，服务业对经济增长的贡献率达55.6%。华耀城、崇盛国际、丽波酒店等项目推进顺利，衡缘、白沙洲物流园加快建设。融资性担保公司、小贷公司实现县域全覆盖。我市成为全国32个宽带骨干网重要节点城市之一。耒阳被评为全国休闲农业与乡村旅游示范县，石鼓书院、蔡伦竹海获评4A级景区，乡村生态旅游、中俄红色旅游合作等节会活动成功举办，全市接待国内外游客人次、实现旅游总收入分别增长14.2%、16%。

二是突出抓改革开放。重点改革实现突破。395家市属国有企业改制圆满完成。市县政府机构改革全面到位，合并乡镇工作基本完成，合并建制村工作进展顺利。在全省率先推出商事制度“改革套餐”，全面启动企业“三证合一”登记制度改革。县级公立医院综合改革稳步推进，涉改的18家县级公立医院全部实行药品零差价销售。金融服务“三农”在全国创造“衡阳经验”，农村“五权”抵押贷款余额达15.4亿元。农村土地承包经营权确权登记颁证进入实质性测绘阶段。国有林场改革全面完成。创新创业活力强劲。建立省级产业技术创新战略联盟和省级大学生创业园各3家，新增创业主体2.4万个。耒阳获评国家知识产权试点城市，衡阳运输机械有限公司被评为国家品牌培育示范企业，特变电工衡变公司被评为国家技术创新示范企业，中国科学院湖南技术转移中心衡阳中心正式挂牌。对外开放成效显著。新签约重大项目90个，科大讯飞、神州数码等高新技术企业及万达、卜蜂莲花、唯品会等知名商业品牌落户衡阳。全市实际利用外资10.25亿美元，增长14%；实现进出口总额32亿美元，增长10%。

三是突出抓城乡统筹。外部交通更加顺畅。湘江土谷塘航电枢纽一期蓄水发电，衡阳港松木港区建成投运，怀邵衡铁路和娄衡高速、南岳高速东延线加快建设。中心城区扩容提质。酃湖、东洲岛片区完成征地拆迁，三江六岸风光带建设及幸福河改造推进顺利。船山东路、二环路、三环路建设及107国道城市化改造有序推进。蒸湘南路延伸线建成通车，船山西路延伸线加快建设。县城面貌明显改观。衡南云集大道等8条主干道完成提质改造，衡阳县城区“百园行动计划”和拆围透绿活动深入开展，衡山毛泽建公园加快建设，衡东河西新区和城北片区开发扎实推进，祁东成为全省新型城镇化试点县，常宁一环线基本贯通，耒阳南北正街完成提质改造，南岳统筹城乡发展示范区建设进展顺利。城乡环境大力改善。开启数字城管新模式，占道经营、渣土运输、社区环卫等专项整治扎实推进。建成公路超限超载检测站10个，车辆超限超载率下降到0.34%。9个国、省考核污水处理厂建成投运，城市生活垃圾焚烧发电厂、餐厨废弃物无害化处理中心加快建设。中心城区社区整合工作全面完成，“三清三建”行动有序推进。

四是突出抓民生改善。认真办好民生实事。新增城镇就业7.08万人，新增农村劳动力转移就业6.3万人。机关事业单位养老保险制度改革和全民参保登记稳步推进，五大保险参保人数达803.76万人次。企业退休人员基本养老金实现“十一连调”。省内异地就医实现即时结算。城乡低保人均月补助标准分别提高到276.5、118.5元。开工建设公租房1.5万套，城市棚户区改造2.78万套。解决40.68万农村人口饮水不安全问题。395个贫困村实现驻村帮扶全覆盖。完成农村公路通畅工程456.6公里，通畅率为100%，行政村客班车通达率达97.8%。农村信息基础设施建设“三年行动计划”顺利实施。全面发展社会事业。完成50所合格学校建设、115所农村中小学改造，扎实推进城区小学化解“大班额”三年行动计划，启动职业教育产教融合基地建设。新农合参合率达99%，基本药物制度实现基层全覆盖；人口和计划生育工作平稳推进，在全省率先实行孕产妇产前筛查全免费。市图书馆主体竣工，村镇、社区文化活动中心基本实现全覆盖；市体育馆建成投用，市十运会成功举办。着力坚守底线工作。全市没有发生较大以上安全生产事故。河道管理和保洁全面加强，城区非法砂场、搅拌站全部取缔到位，规模养殖场无害化处理实现全覆盖，城区空气质量达标率达91%。深入开展信访“五零”专项行动，大力创建“平安衡阳”，农村“天网工程”经验在全省推介。严厉打击非法集资，守住了不发生系统性和区域性金融风险的底线。

五是突出抓自身建设。取消和下放116项行政审批事项，行政审批精简率达60.2%，全面取消非行政许可审批事项，制定发布“三清单一目录”，全面推进“两集中、两到位”改革，市政务服务中心新址投入使用。扎实推进依法行政，建立完善政府法律顾问制度，建市以来首获地方立法权。自觉接受人大法律监督和工作监督、政协民主监督、社会各界监督，充分吸纳各民主党派、工商联和无党派人士意见、建议，办理市人大代表议案1件、建议355件，办理市政协提案447件。扎实开展“三严三实”专题教育，干部作风持续好转。全面落实党风廉政建设责任制，严格遵守中央“八项规定”，加大腐败案件查处力度，全市纪检监察机关立案1314件，查处1062人。纠错问责机制不断完善，行政监察和审计监督力度持续加大，实现对征地拆迁安置资金的审计全覆盖。

支持国防和军队现代化建设。国防动员和后备力量建设质量提高，优抚安置和民兵预备役建设、人防工作得到加强。双拥共建取得新成效，军地融合发展呈现新局面。

各位代表!

以上成绩的取得，是市委坚强领导、总揽全局的结果，是全市上下团结奋斗、顽强拼搏的结果，是社会各界关心支持、共同努力的结果。在此，我代表市人民政府，向全市人民，向各民主党派、人民团体，向驻衡部队、武警官兵、政法干警，向中央、省驻衡单位，向关心支持衡阳建设发展的各界人士，表示衷心的感谢!

各位代表!

在肯定成绩的同时，我们也清醒地认识到，政府的工作与人民的期待还存在一定差距，发展还面临不少的困难和问题：一是受宏观经济形势严峻、结构调整、清理历史虚增等因素影响，“十二五”规划和2015年确定的部分经济指标没有完成。二是经济发展动力尚未完成转换，农业产业特色不鲜明，工业稳增长基础不牢，现代服务业潜力有待挖掘。三是发展不平衡、不协调、不可持续问题仍然突出，城乡、区域、行业之间的差距较大，县域经济发展总体水平较低。四是扶贫攻坚任务艰巨，部分群众生活还比较困难，群众反映的一些突出问题没有得到彻底解决。五是政府职能转变有待加强，政务环境、亲商氛围有待改善提升。对于这些问题和不足，我们将切实采取措施，努力加以解决。

二、“十三五”时期总体思路及目标任务

“十三五”时期是全面建成小康社会的决胜阶段，是实现升级发展的关键时期。我市经过“十二五”时期的快速发展，产业体系不断完善，发展平台持续夯实，综合交通日趋完备，城市发展提质提速，综合实力明显增强，具备了加速崛起的良好基础，正处于全领域推进开发开放的黄金时期。我们要准确把握战略机遇期内涵的深刻变化，有效应对各种风险和挑战，积极对接国家“一带一路”和长江经济带战略，着力在优化结构、增强动力、化解矛盾、补齐短板上实现突破性进展，坚定不移地为实现第一个百年目标而努力奋斗。

今后五年，全市经济社会发展的指导思想是：高举中国特色社会主义伟大旗帜，全面贯彻党的十八大和十八届三中、四中、五中全会精神，以马克思列宁主义、毛泽东思想、邓小平理论、“三个代表”重要思想、科学发展观为指导，深入贯彻习近平总书记系列重要讲话精神，坚持“四个全面”战略布局，贯彻创新、协调、绿色、开放、共享发展理念，坚持发展第一要务，坚持以提高经济发展质量和效益为中心，围绕建设“工业重镇、文化名城、旅游胜地、宜居家园”目标，深入实施“开放带动、基础先行、兴工强农、创新发展”战略，统筹推进经济建设、政治建设、文化建设、社会建设、生态文明建设和党的建设，确保如期全面建成小康社会，找回全省第二大城市的历史荣光。

“十三五”时期，全市要努力实现以下目标：

一是经济保持中高速增长。地区生产总值年均增长9%左右，2020年总量达到4000亿元(按2015年可比价计算)，人均GDP超过8500美元。财政总收入年均增长10%，2020年总量达到420亿元。固定资产投资、社会消费品零售总额、进出口总额年均分别增长16%、12%、15%。规模工业增加值年均增长9.5%，2020年战略性新兴产业增加值比2015年翻一番。

二是发展方式加快转变。工业化和信息化深度融合发展，打造电子信息、高端装备制造、有色金属加工三大千亿产业集群。农业现代化加快推进，建设优质农产品主产区，2020年农产品加工业总产值突破2300亿元。现代服务业加快发展，积极打造国际旅游目的地城市，加快建设中国南部物流中心城市、全国综合运输服务示范城市和区域性金融服务中心。创新驱动能力显著增强，2020年科技进步对经济增长的贡献率提高到60%。

三是城乡统筹扎实推进。成功创建全国文明城市。到2020年，市中心城区建成区面积达160平方公里、常住人口达170万人，全市城镇化率提高到56%。扎实推进交通、产业、配套设施融城战略，形成中心城区和周边县(市)协同发展格局，城区经济与县域经济良性互动，城乡空间布局合理、产业分工明确、发展要素共享，城乡差距持续缩小。

四是生态环境质量明显提升。国家生态文明先行示范区建设扎实推进。主体功能区制度严格落实，生态保护与环境治理全面加强。推动低碳循环发展，绿色生产方式和消费方式加快形成，到2020年，全市森林覆盖率超过50%，城市建成区绿化覆盖率达42%，空气质量优良天数比率超过90%，城镇污水集中处理率达90%，城镇生活垃圾无害化处理和资源化利用率达90%。能源资源开发利用效率大幅提高，建设用地得到有效控制，全面完成节能减排目标任务。

五是社会民生持续大力改善。收入差距不断缩小，中等收入人口比重不断上升，居民人均可支配收入年均增长9%以上。2018年前实现农村贫困人口整体脱贫。城镇调查失业率控制在6%以内。到2020年，劳动年龄人口平均受教育年限10.7年，每千名老年人口养老床位数达到50张；县级以上城区保障性住房覆盖面达23%，农村危房改造覆盖面为100%；公共文化服务体系基本建成，文化及相关产业增加值占GDP比重超过5%，文化遗产得到有效保护，成功申报国家历史文化名城。政府工作报告

三、2016年工作安排

完成“十三五”各项目标任务，全面建成小康社会，必须扎实做好2016年各项工作，确保开好局、起好步。

今年全市经济和社会发展的主要预期目标是：地区生产总值增长9%以上，财政总收入增长10%以上，固定资产投资增长18%以上，社会消费品零售总额增长12.5%，进出口总额增长15%，规模工业增加值增长8.5%左右，城乡居民人均可支配收入分别增长9%、9.5%，全面完成省里下达的节能减排、安全生产等约束性指标任务。

实现上述目标，要切实做好以下几个方面的工作：

(一)坚持创新驱动，着力提高发展质量和效益

全面推进科技创新。在先进装备制造、电子信息、生物医药等重点领域布局重大科技专项，支持研发一批具有自主知识产权的核心技术，实施知识产权和品牌发展战略，实行企业技术需求和科技成果转化清单制度。大力培育高新技术企业，重点扶持天雁机械、南岳电控攻克关键技术，支持二七二、共创光伏等加强技术研发和产品创新。支持企业与高校、科研院所共建企业技术研发中心、重点实验室，年内新建省级企业技术研发中心2家以上。加快湘南自主创新基地、启迪科技园、南华大学科技园规划建设，推动华为公司在衡创立联合创新中心。

大力激发“双创”活力。做大做强创客经济，启动创客平台建设，打造全市科技企业孵化器，建设中小微企业创业基地。大力扶持非公有制经济发展，引导符合条件的个体工商户转型为企业，加快构建现代市场体系，着力培育壮大市场主体。推进创新创业资助工程、大学生创业引领计划，鼓励高校、科研院所的专业技术人员离岗创业。建设一批农业农村创业园区，引导能人和农民工回乡创业。

努力增强发展动力。充分发挥政府投资的带动作用，扎实抓好500个以上、年度投资1000亿元以上的重点项目建设。支持社会资本以PPP等模式参与公共基础设施建设。全面优化投资结构，引导更多资金投向技术创新、环境保护、产业升级、公共服务等领域。大力培育新兴消费热点，不断挖掘服务消费、信息消费、农村消费、绿色消费、时尚消费和品质提升型消费潜力。加快建设人才强市。实施专业技术人才开发工程，加强域外智力引进。实施高技能人才振兴工程，培育一批“衡阳名师工匠”。实施人才培养提升计划，大力培养新生代企业家。实施人才优先发展战略，鼓励人才以专利、技术、知识等投资入股。为来衡工作的高层次人才提供优质的教育、医疗等资源。

(二)全面深化改革开放，进一步激发经济发展活力

持续推进重点改革。从供需两侧同时发力，推进供给侧结构性改革，提升有效供给能力；加强需求侧管理，挖掘释放内需潜力。稳步推进财税体制改革，落实减税政策，推进财政资金统筹使用。深化国有企业改革，盘活改制企业国有资产，不断完善国有企业法人治理结构，建立现代企业制度。深化县级公立医院综合改革，积极探索现代医院管理机制。加快不动产统一登记制度改革，实现统一登记发证。深化城区、园区、文化、生态文明等体制改革。

提升开放型经济发展水平。拓展综保区功能，支持综保购发展。推进湘江衡阳至株洲段2000吨级航道、铁路口岸、大浦通用机场建设。支持“三自三高”企业的产品、装备产能、工程承包、服务外包出国出海，开拓国际市场。鼓励外资以参股并购等方式参与企业改组改造和兼并重组，优化配置存量企业资源。

加大招商引资力度。抢抓产业转型机遇期，创新承接产业转移模式及服务方式，着力引进行业领军企业、科技先导企业，吸引更多企业来衡建立集团总部、区域总部和研发、采购中心。大力开展专题招商活动，积极推行小分队招商、产业链招商，引导衡商回故乡、建家乡。

(三)加快推进新型工业化，打造工业经济升级版

壮大战略性新兴产业。对接“中国制造2025”关键领域，抢占智能制造技术和市场制高点。发挥富士康、欧姆龙等企业的带动效应，打造电子信息产业集群。强化特变电工、天雁机械等企业的龙头牵引作用，培育高端装备制造产业集群。扩大中国五矿的铜精深加工优势，建立有色金属加工产业集群。加大技术研发和产业化运用，支持南岳生物、紫光古汉、恒生制药打造现代生物制药产业。做大做强新能源、新材料产业。

推进传统产业转型升级。积极利用先进适用技术、先进工艺、对标管理改造提升传统优势产业，促进传统产业向价值链中高端迈进。运用先进节能环保技术、工艺和装备，全面推进传统制造业两型化改造，加快汽车零部件等传统产业提质升级，采取兼并重组、债务重组和破产清算等方式，积极稳妥化解钢铁、化工、建材等行业过剩产能。开展质量品牌提升行动，推进纺织、食品、酿酒等消费品行业质量品牌建设。

加快园区扩容提质。深入推进创新创业园区“135”工程和“五大平台”建设，打造工业园区发展升级版。启动建设雨母智慧生态发展区，加快白沙洲工业园东阳渡新扩区域基础设施建设，支持打造建滔电子信息产业园，推动水口山经开区升格为国家级经开区；支持衡山科学城建设高端研发制造基地，加快大数据库、互联网金融众筹服务平台建设。提高各县(市)工业园区配套能力和管理水平，强力推进西渡高新区、归阳工业园基础设施及企业建设，加快大浦工业园、大市循环工业园等循环经济园区建设。

(四)完善农业产业化发展体系，加快农业现代化建设

构建农业产业新体系。提质加速建设8万亩高标准农田，稳固粮食主产区地位，粮食总产量稳定在330万吨以上。抓好以水库除险加固、灌区续建配套和节水改造、“五小”水利为重点的农田水利建设。加快构建十大现代农业产业体系，推进城区3万亩蔬菜标准园和8大特色蔬菜基地建设，发展壮大特色农产品基地，抓好衡阳国家现代农业科技园、华夏湘江农业产业示范园等十大农业园区建设。深入实施农产品加工业振兴规划，扶持农产品精深加工龙头企业发展，推进农业标准化生产和品牌建设，支持农产品地理标志认证，打造全国知名品牌。

创新农业经营新模式。基本完成农村土地承包经营权确权登记颁证工作。稳定农村土地承包关系，加快土地流转，发展多种形式的适度规模经营。深入实施“稻稻油”攻坚工程和油菜全程机械化促进行动，推进“十百千万”现代农业发展工程。加快培育新型农业经营主体和职业农民。实施休闲农业示范区建设工程，因地制宜拓展农业功能。全年新增省级休闲农庄10家、新型农业经营主体1000个。

拓展农业服务新空间。推进政府购买农业公益性服务，推广订单、合作、托管等全过程社会化服务模式。加快乡镇农业服务中心建设。加强农业科技服务，完善现代农业科技创新推广体系。推进农业信息服务，加强村级信息服务站建设，加快实现农产品生产、加工、储藏、运输和销售全过程信息化。规范农村市场服务，推进农超、农社对接，支持农资配送专业公司和农村电商发展，健全农副产品现代购销网络，促进农产品高效流通。加大金融服务“三农”力度，积极创新农村金融产品和抵押信贷模式，探索成立面向“三农”的政策性担保公司，大力发展农业保险。

(五)推进现代服务业发展，加快构建区域性服务业中心

壮大专业化生产性服务业。加快衡缘、白沙洲、铁路物流园及松木水路口岸建设，开拓云集空港物流市场，大力发展第三方物流和智能物流，提高物流行业标准化服务水平。加速快件中心建设，培育建设专业型外贸服务基地。加快发展总部经济、会展经济等新型商业业态，抓好华耀城、唯品会等项目建设。

发展高品质生活性服务业。积极引进集写字楼、大型购物广场、星级酒店等于一体的商业综合体，加快万达商业广场、崇盛国际、丽波酒店建设。改造提升老旧市场，发展新兴专业市场，打造具有区域竞争力的专业市场群。丰富健康、家政、养老等服务产品供给，支持医养结合发展养老产业，大力发展社区日间托管养老、居家养老，推进政府购买养老服务。规范发展教育培训、休闲娱乐、住宿餐饮等服务业。加快文化服务业发展，支持大型俱乐部连锁经营，培育大型文体休闲服务企业。

打造国际旅游目的地城市。实施旅游精品建设工程，提质升级南岳衡山核心景区，推动南岳旅游转型升级。加强雨母山、四方山景区及天堂山、四明山国家森林公园建设。支持石鼓书院、东洲岛、廻雁峰打捆创建5A级景区，推动3A级景区创建4A级景区。培育新兴旅游业态，打造衡阳湘江旅游经济带。推进乡村旅游富民工程，发展以体验型休闲农业为补充的现代都市旅游业。打造2-3条精品旅游线路。力争全年接待国内外游客突破5500万人次，实现旅游总收入350亿元。

培育壮大金融服务业。加快农信社改革发展，完成农信社改革组建农商行任务。创新金融产品和服务，优化信贷结构，力争年内实现社会融资300亿元，其中新增直接融资100亿元。壮大金融服务主体，引进大型金融机构落户衡阳。加快政府投融资公司转型发展。推进湖南机油泵、南岳电控上市，支持企业在“新三板”、区域性股权交易市场挂牌，提高直接融资规模和比重。规范各类融资行为，加强风险监测预警和处置，坚决守住不发生系统性和区域性金融风险的底线。

(六)大力推进新型城镇化，加快城乡一体化进程

提升中心城区辐射力。围绕打造全省次中心城市目标，拓展城市发展空间，加快建设来雁新城、滨江新区、酃湖片区、东洲岛片区、白沙新城。完善城市路网结构，加快怀邵衡铁路、娄衡高速、南岳高速东延线和二环路、北三环建设，拉通船山东路，完成107国道城市化改造，加快国省干线公路提质改造。推进管廊城市、海绵城市建设，完善无障碍通道、地下人行通道、地下管沟和雨污分流系统，加快雁栖湖综合改造治理，完成25条主要道路两厢绿化提质。加快社区“两化两体系”建设，全面推进中心城区社区“三清三建”行动。

加速县城建设发展。各县(市)按照“因地制宜、优势优先”要求，差异化打造特色主导产业，实现产城一体发展。推动县城扩容提质，加快基础设施完善与配套，提高城市管理水平。加快衡南融城步伐，加速衡阳县城蒸水风光带建设，推进衡山开云新城、衡东城东滨水区、祁东玉泉新区、常宁青阳新区及南岳西片区开发，加快耒阳“三纵三横一环线”干线公路建设。

推进小城镇建设。加快交通融城，促进市区与周边卫星城镇的互联互通，确保船山西路延伸线建成通车，启动衡州大道西延工程。加快产城融合，推动建设产业群、经济带和新型特色城镇带。探索建立城乡统一的建设用地市场，乡镇土地增值收益全部用于小城镇基础设施建设。积极创建文明村镇和“秀美村庄”，加强新农村示范片建设，加强风景名胜区及传统村落保护。

提升城市管理水平。从群众需求出发完善城市规划，完成总规修改报批和乡镇规划编制，坚决控制城市规划区内违法违规建设。全面推进“八城同创”，深化城市管理工作市场化改革。加快推进电动摩托车上牌等城市管理地方立法工作。推动客运班线城际公交化改造，加大出租车行业监管，建设高新区公共自行车租赁系统，提升城市公共交通服务水平。巩固殡葬改革成果，加大烟花爆竹限放工作力度。加快户籍和居住证制度改革，推进农民工市民化，实现住房存量资源的优化配置，有序消化现有房地产库存，促进房地产业健康发展。

(七)实施信息化发展战略，建设智慧衡阳

全面夯实信息基础设施。加快实施“宽带中国”战略，建设光网城市和无线城市。大力推进农村信息基础设施建设“三年行动计划”，统筹城乡区域信息网。加快“三网融合”进程，推进电信网和广播电视网基础设施共建共享。促进“数字衡阳”市县一体化和部门信息共享，加快衡阳数据中心、中兴网信全球共享服务中心建设。

扎实推进“互联网+”行动。支持特变电工、衡钢、合力叉车等龙头企业建设自动化车间和智能工厂。加快衡山科学城互联网装备及新材料企业建设，推进电子信息制造业、物联网等产业发展。构建电子商务产业链，加快打造省级电子商务产业集聚示范基地和湘南区域电子商务中心。

提升信息化应用水平。实施衡阳大数据行动计划，促进大数据解决方案在公共服务、城市管理、教育及产业发展等方面的应用。完善“12345”政府服务热线和政务信息资源共享交换平台，推动政府机关和事业单位数据业务外包，构建覆盖全市的电子政务网络，集约建设政府及部门网站群，推动政府实体行政办事大厅向网上迁移。

(八)着力改善生态环境，建设生态文明城市

加大生态建设力度。抓好国家生态文明先行示范区建设。实施绿化增量提质攻坚行动，加强通道绿化建设，拓展城乡绿化空间，全市森林覆盖率超过47%。加快洣水国家湿地公园和幸福河、横江、萱洲湿地公园建设，提升湿地保护管理能力。推进防洪保护圈、河湖连通工程建设。加强河道保洁与采砂管理，持续推进矿山生态修复治理工程，大力预防和治理水土流失。

加大污染防治力度。站在更高起点推进湘江流域保护和治理第二个“三年行动计划”。加强固体废物污染防治和水口山重金属污染治理。推进垃圾场综合整治，建立城乡一体的垃圾收容体系，确保城市生活垃圾焚烧发电厂、餐厨废弃物无害化处理中心建成营运。建成松木、角山、酃湖污水处理厂，加快乡镇污水处理厂建设。启动市污泥处置中心建设。继续开展农村环境和卫生综合整治，实施农药、化肥使用量零增长行动。

节约集约利用资源。实施强制性能耗标准，严格执行固定资产投资项目节能评估审查制度，强力淘汰落后产能，健全排污权交易制度。坚持最严格的耕地保护制度，加大闲置土地清理力度。强化用水总量、用水效率、水功能区限制纳污“三条红线”控制。推广绿色建筑和建材。鼓励和支持可再生能源发展，大力发展生产、流通环节的能源经济。

(九)统筹发展社会事业，不断增进人民福祉

全力办好民生实事。落实就业优先战略，实施更加积极的就业政策，以创业带动就业。推行终身职业技能培训制度，全年新增城镇就业6万人，新增农村劳动力转移就业5.8万人。巩固提升农村饮水安全工程。完成900公里农村通客班车路段加宽改造任务。加快安置房建设和中心城区商品房产权办理遗留问题处理进度。全面推进农网升级改造工作。

加快建设教育强市。推进城区中小学化解“大班额”攻坚行动，积极推进义务教育合格学校建设，实施第二轮学前教育“三年行动计划”，加快普及高中教育。规范、扶持民办教育发展，加快职业教育产教融合基地建设。大力发展继续教育，办好特殊教育。整体推进教育信息化建设。加大学校债务化解力度。坚持依法治教，推进学校自主办学。强化教师队伍建设。

着力建设文化名城。加强社会主义核心价值体系建设。加快构建现代公共文化服务体系，大力推进市博物馆、科技馆、规划馆建设，加快实施“户户通”“村村响”“无线数字化覆盖”等文化惠民工程。加强对艺术创作生产的引导，繁荣文学艺术、新闻出版、广播影视事业。做好衡山皮影戏、祁东渔鼓、常宁版画等文化遗产的传承与保护，加大文物古迹保护力度，扎实推进国家历史文化名城申报工作，建设湖湘文化传承与创新示范区。

积极建设健康衡阳。完善新农合制度，巩固基本药物制度实施成果。加强以全科医生为重点的基层卫生人才培养，加快乡镇卫生院、村卫生室标准化建设。扶持中医药事业发展。深化“平安医院”创建，依法惩处涉医犯罪和“医闹”行为。实施食品安全战略，健全食品药品质量安全治理、保障体系，让人民群众吃得放心、喝得安全。大力发展体育事业，深入推进全民健身运动，扎实做好省十三届运动会、省十届残运会筹备工作。

完善社会保障体系。健全社会保险体系，实施城乡居民大病保险制度，完成全民参保登记，推进被征地农民社会保障工作，加强医疗保险城乡统筹。健全社会救助体系，全面开展重特大疾病医疗救助，加快推进最低生活保障制度城乡统筹。推行货币化安置，启动政府购买棚改服务，全年开工建设保障性安居工程5万套以上，公租房资源重点向基层教师、医生和公务员倾斜。

提高居民收入水平。健全科学的工资水平决定、正常增长、支付保障机制，完善企业工资集体协商制度，落实最低工资保障制度。完善再分配调节机制，增加低收入劳动者收入，扩大中等收入者比重，多渠道增加居民财产性收入。在县级及以下机关推行公务员职务与职级并行、职级与待遇挂钩制度。

扎实推进扶贫攻坚。全面实施精准扶贫战略，更加注重对特定人群特殊困难的精准帮扶，举全市之力，全面落实十大扶贫措施，产业扶贫惠及贫困人口50%以上，推进驻村、联村和结对帮扶全覆盖，年内完成扶贫开发投入2.5亿元以上，完成395个驻村帮扶贫困村D级危房户和无房户的改造建设任务，实现7万贫困人口脱贫。

促进人口均衡发展。坚持计划生育基本国策，完善利益导向机制，落实“全面二孩”政策。完善困境儿童福利制度，构建农村留守儿童关爱服务体系，促进妇女儿童事业全面发展。提升残疾人服务保障水平。加快慈善事业发展。(十)全面推进依法治市，提升民主法治建设水平

提升全民法治意识。不断加强精神文明建设，全面实施公民道德建设工程，深化群众精神文明创建活动。推进“七五”普法，落实“谁执法、谁普法”责任制，加快推进法治教育全覆盖，增强全社会学法尊法守法用法观念，不断提升群众法治思维和法治意识。依法管理民族、宗教事务和场所。

发展社会主义民主政治。健全权力运行制约和协调机制，坚持重大事项请示报告制度。自觉接受人大法律监督和工作监督、政协民主监督，以及舆论监督、群众监督，认真办理各级人大代表议案、建议及政协提案。全面完成建制村合并工作，做好全市村(居)委会同步换届工作。

努力创新社会治理方式。加快建设覆盖城乡的法律服务体系，完善社会矛盾排查预警和调处化解机制。发挥居民主体作用，构建全民共建共享的社会治理模式。推进信访工作法治化改革，健全涉法涉诉信访依法终结机制。继续抓好“平安衡阳”建设，扎实推进“五打四建”，强化治安防控体系建设。不断健全公共安全体系，提高公共突发事件防范和处置能力。完善和落实安全生产责任和管理制度，确保不发生较大以上安全生产事故。强化防灾减灾能力建设。加强网络信息安全管理。

(十一)持续加强自身建设，打造人民满意政府

加大政府改革力度。全面深化“两集中、两到位”改革，深入推进行政审批制度改革，加大简政放权力度，加快政府职能转变。探索委托乡镇一级行使部分县级行政执法权限。依法有序推进园区“封闭运行”，赋予园区更多发展自主权。深化政务公开，加强政务服务，不断降低制度性交易成本，努力营造一流的亲商环境。完善政府绩效评价机制。

深入推进依法行政。继续落实“三清单一目录”制度。建立重大决策终身责任追究制度及责任倒查机制。深化行政执法体制改革，完善行政执法程序，大力推进跨部门跨行业综合执法、联合执法，加强行政执法监督。完善政府法律顾问制度。支持监察、审计、财政、法制等部门开展监督工作。

守住廉政底线。全面落实廉洁从政各项规定，深入推进惩治和预防腐败体系建设。认真落实党风廉政建设主体责任和监督责任，落实“一岗双责”，始终保持惩治腐败高压态势。持续巩固党的群众路线教育实践活动和“三严三实”专题教育成果，全面净化政风行风，努力建设廉洁政府。

推进国防建设。坚决支持深化国防和军队改革，做好国防动员、民兵预备役和人防工作，深入开展双拥活动，抓好退役军人安置工作，加强全民国防教育和后备力量建设，加快建设东阳半岛军民融合产业园，推动经济建设和国防建设融合发展。

各位代表，“十三五”宏伟蓝图已经绘就，历史重任落在我们肩上。让我们紧密团结在以习近平同志为总书记的党中央周围，在市委的正确领导下，紧紧依靠全市人民，解放思想、开拓创新、扎实工作，为推动衡阳转型跨越发展、如期全面建成小康社会而努力奋斗!
